# SWI/SNF complexes are required for full activation of the DNA-damage response

**DOI:** 10.18632/oncotarget.2715

**Published:** 2015-01-06

**Authors:** Stephanie L. Smith-Roe, Jun Nakamura, Darcy Holley, Paul D. Chastain, Gary B. Rosson, Dennis A. Simpson, John R. Ridpath, David G. Kaufman, William K. Kaufmann, Scott J. Bultman

**Affiliations:** ^1^ Department of Genetics and Lineberger Comprehensive Cancer Center, University of North Carolina, Chapel Hill, NC, USA; ^2^ Department of Environmental Sciences and Engineering, University of North Carolina, Chapel Hill, NC, USA; ^3^ Department of Pathology and Laboratory Medicine, University of North Carolina, Chapel Hill, NC, USA; ^4^ Department of Biomedical Sciences, William Carey University, Hattiesburg, MS; ^5^*Current address*: Division of the National Toxicology Program, NIEHS, Research Triangle Park, NC

**Keywords:** BRG1, BRM, tumor suppression, DNA damage response, chemotherapeutics

## Abstract

SWI/SNF complexes utilize BRG1 (also known as SMARCA4) or BRM (also known as SMARCA2) as alternative catalytic subunits with ATPase activity to remodel chromatin. These chromatin-remodeling complexes are required for mammalian development and are mutated in ~20% of all human primary tumors. Yet our knowledge of their tumor-suppressor mechanism is limited. To investigate the role of SWI/SNF complexes in the DNA-damage response (DDR), we used shRNAs to deplete BRG1 and BRM and then exposed these cells to a panel of 6 genotoxic agents. Compared to controls, the shRNA knockdown cells were hypersensitive to certain genotoxic agents that cause double-strand breaks (DSBs) associated with stalled/collapsed replication forks but not to ionizing radiation-induced DSBs that arise independently of DNA replication. These findings were supported by our analysis of DDR kinases, which demonstrated a more prominent role for SWI/SNF in the activation of the ATR-Chk1 pathway than the ATM-Chk2 pathway. Surprisingly, γH2AX induction was attenuated in shRNA knockdown cells exposed to a topoisomerase II inhibitor (etoposide) but not to other genotoxic agents including IR. However, this finding is compatible with recent studies linking SWI/SNF with TOP2A and TOP2BP1. Depletion of BRG1 and BRM did not result in genomic instability in a tumor-derived cell line but did result in nucleoplasmic bridges in normal human fibroblasts. Taken together, these results suggest that SWI/SNF tumor-suppressor activity involves a role in the DDR to attenuate replicative stress and genomic instability. These results may also help to inform the selection of chemotherapeutics for tumors deficient for SWI/SNF function.

## INTRODUCTION

In eukaryotic cells, genomic DNA is packaged as chromatin, and the nucleosome is the most fundamental unit. Although nucleosomes help compact the genome and maintain its organization, they are an impediment to transcription, DNA replication, and DNA repair. To counteract nucleosomes and facilitate these essential processes, SWI/SNF chromatin-remodeling complexes have been evolutionarily conserved from yeast to humans [1–3]. Recruited by pioneer transcription factors to specific sites in the genome, SWI/SNF utilizes the energy of ATP hydrolysis to slide or evict nucleosomes [1–3].

Mammalian SWI/SNF complexes utilize either BRG1 (also known as SMARCA4) or BRM (also known as SMARCA2) as alternative catalytic subunits with DNA-dependent ATPase activities and contain 8–11 additional subunits (often referred to as BAFs) [1–3]. Based on gene-targeting experiments in mice, SWI/SNF complexes are required for embryonic development [2, 3]. SWI/SNF complexes are important for human development as well. For example, *BRG1* and *BRM* mutations (as well as *BAF250A/ARID1A* and *BAF250B/ARID1B* mutations) are responsible for Coffin-Siris and Nicolaides-Baraitser syndromes which have similar phenotypic spectrums that include intellectual disability, altered craniofacial features, and distal limb anomalies [4–6]. These mutations occur *de novo* and are heterozygous, which implies that these SWI/SNF subunits are extremely dosage sensitive.

SWI/SNF complexes also function as tumor suppressors based on somatic, loss-of-function mutations in human tumors [7]. Exome-sequencing projects consistently identify recurrent SWI/SNF mutations in primary human tumors of diverse origin. Meta-analyses of these data indicate that ~20% of all human tumors have a mutation in SWI/SNF, which is among the highest incidence of any tumor suppressor and approaches the *TP53* mutation frequency of 26% [8, 9]. The majority of SWI/SNF mutations occur in the *BRG1/SMARCA4* catalytic subunit and *BAF250A/ARID1A, BAF250B/ARID1B,* or *BAF180/BRM1*, which contain ARID and bromodomains that bind to DNA and acetylated histones, respectively. Several genetically-engineered mouse models support the human data. For example, although *Brg1* constitutive null homozygotes are embryonic lethal, heterozygotes develop mammary tumors without exposure to any oncogenic agents [10, 11]. In this model, BRG1 is a haploinsufficient tumor suppressor as the tumors do not undergo loss of heterozygosity (LOH) and the wild-type allele is not silenced.

An important challenge is to understand the mechanism of SWI/SNF-mediated tumor suppression. SWI/SNF complexes have been studied primarily in the context of transcriptional regulation, and several tumor-suppressor and proto-oncogene targets have been identified. For example, BRG1 and SNF5/BAF47 bind to the promoters of the *p16^INK4a^* and *p21^CIP/WAP1^* cyclin-dependent kinase (CDK) inhibitors and activate their expression in tumor-derived cell lines [12–16]. SWI/SNF has also been linked to nuclear-hormone receptor signaling, the hedgehog-GLI pathway, RB and E2F1, CD44 and c-MYC *in vitro* [7], but it is currently unclear whether any of these targets are relevant for tumor suppression *in vivo*.

Because SWI/SNF complexes are chromatin remodelers, they are undoubtedly important for DNA-templated processes other than transcription such as DNA replication and DNA repair. However, these fundamental processes have not been investigated adequately due to technical limitations. In contrast to transcriptional studies, where ChIP and ChIP-seq experiments have documented the occupancy of BRG1 and other SWI/SNF subunits at enhancers and promoters of target genes in various cell types, this methodology is not appropriate for DNA replication/repair studies. The fact that DNA replication and DNA repair occur at different genomic sites in different cells within a population at any given time results in a lack of discrete peaks and precludes ChIP and related (e.g., FAIRE) methods. However, consistent with the proposed role(s) of SWI/SNF in DNA replication/repair, we previously demonstrated that BRG1 co-IPs and co-localizes with components of the DNA replication machinery such as the GINS complex, PCNA (proliferating cell nuclear antigen), and TOPBP1 (topoisomerase II-binding protein 1) during S phase [17]. More importantly, this study also demonstrated that perturbation of both SWI/SNF catalytic subunits (BRG1 and BRM) in mouse embryos or D98 tissue-culture cells results in a ~50% reduction in the efficiency of replication fork progression, which phenocopies RNAi-mediated knockdowns of Chk1, timeless, and claspin [18, 19]. This finding suggests that there might be a functional relationship between SWI/SNF and the DNA-damage response (DDR) or the replication fork protection complex (RFPC) and has implications for tumorigenesis because collapsed replication forks are a major driver of genomic instability [20]. Indeed, our *Brg1^+/null^* mouse model of breast cancer has mammary tumors with extensive copy-number gains (i.e., duplications and amplifications) and losses (i.e., deletions) [11].

The DDR is a cellular surveillance system that senses DNA damage and elicits an appropriate response that includes DNA repair or apoptosis to prevent genomic instability and cancer [21–24]. The DDR also regulates CDKs and checkpoints to delay or arrest cell-cycle progression and stabilize replication forks until the DNA damage has been bypassed or repaired, and this is crucial to prevent genomic instability. Not surprisingly, mutations of human DDR genes cause a number of genetic diseases/syndromes and cancer [21]. The DDR, which has been conserved from yeast to humans, can be divided into two major pathways that respond to different types of DNA damage although there is some overlap. First, the PI3 kinase family member ATM (ataxia telangiectasia mutated) senses double-strand breaks (DSBs) induced by ionizing radiation (IR) and activates many targets including the Chk2 checkpoint kinase and the histone variant H2AX. Second, another member of the PI3 kinase family, ATR (ATM- and Rad3-related), senses excess RPA (replication protein A)-coated ssDNA that arises during S phase because of stalled replication forks. Stalling occurs in response to endogenous lesions and a variety of genotoxic agents (e.g., ultraviolet light, PARP and topoisomerase inhibitors, and aphidicolin) and involves uncoupling of the MCM helicase and DNA Polymerase. Recruitment of ATR to a stalled fork by ATRIP (ATR-interacting protein) binding to RPA kickstarts a DDR signaling pathway mediated, in part, by the replication fork protection complex (RFPC, which includes TIM-TIPIN dimers and claspin) and γH2AX induction, and is transduced by the phosphorylation/activation of the checkpoint kinase Chk1. This culminates in activation of the intra-S phase checkpoint, which inhibits origin initiation and decreases the rate of active replication forks, until the DNA damage is bypassed or repaired [21–24]. The ATR signaling cascade prevents the collapse of stalled forks, which results in DSBs that cannot be repaired by DNA replication restart and can lead to genomic instability.

Considering the importance of SWI/SNF complexes in tumor suppression, it is surprising how little we know about their potential role in the DDR. To address this important issue, we have investigated the DDR in D98 tissue-culture cells because of their ability to survive when BRG1 and BRM are simultaneously depleted using shRNAs [17]. D98 cells are physiologically relevant because they are derived from a cervical carcinoma (they are a HeLa cell subline) [25], and SWI/SNF subunits are mutated or silenced in cervical and uterine/endometrial tumors from mouse models and humans [26–29]. D98 cells are advantageous compared to primary cervical/uterine tumors because they are homogeneous and can be exposed to genotoxic agents in a rigorously controlled manner.

## RESULTS

### SWI/SNF protects against cell lethality induced by certain genotoxic agents that cause DSBs associated with DNA replication but not IR

**Figure 1:**
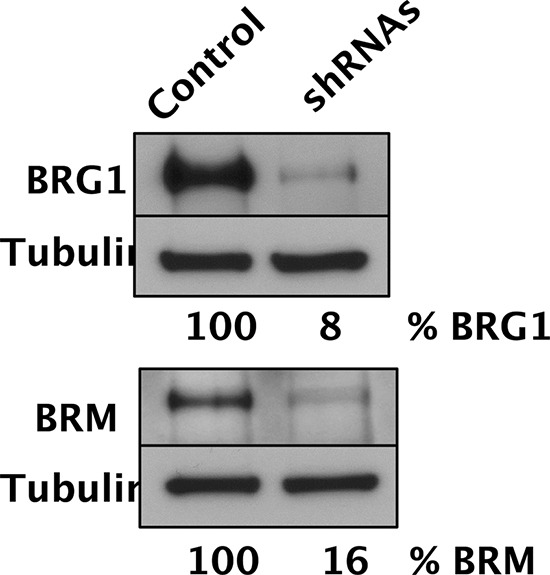
Simultaneous depletion of both SWI/SNF catalytic subunits in D98 cells Western blot analysis of BRG1, BRM, and tubulin loading control of D98 control cells and shRNA knockdown cells. After normalizing protein levels to tubulin, % BRG1 and % BRM refer to the amount of protein remaining in the cells expressing shRNAs compared to the control cell line.

**Figure 2:**
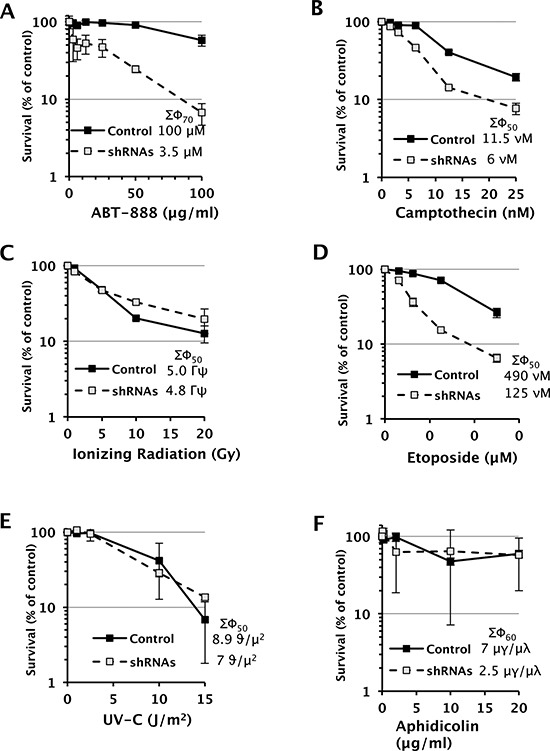
Dose-response survival curves of D98 control cells and shRNA knockdown cells after being exposed to various genotoxic agents Each data point represents the mean ± standard deviation for three replicates. The legend displays the SF50 dose for controls and shRNA cells. SF70 values are provided when the SF50 is not reached in control cells.

To perturb the function of SWI/SNF complexes in D98 cells, the BRG1 and BRM catalytic subunits were simultaneously depleted by constitutive expression of shRNAs. Western blot analyses demonstrated that BRG1 and BRM were depleted to 8% and 16% of wild-type levels, respectively (Fig. 1). Next, we exposed D98 control cells and shRNA knockdown cells to a panel of genotoxic agents. We began with ABT-888, which is a poly(ADP-ribose) polymerase (PARP) inhibitor, because BRG1 co-immunoprecipitates with PARP1 [30, 31]. PARP is a component of the base excision repair (BER) pathway and repairs single-strand breaks (SSBs) that arise during the repair of endogenous base damage such as abasic sites. PARP inhibitors lead to the persistence of SSBs [32]. When encountered by replication forks during DNA replication, SSBs are often converted to double-strand breaks (DSBs). The potential lethality of DSBs is increased dramatically if BRCA1 or other components of homologous recombination repair are mutated or silenced [33, 34]. In addition to interacting with PARP, components of SWI/SNF complexes, including BRG1, interact with BRCA1 [35–37]. Although the interaction between SWI/SNF complexes with PARP or BRCA1 has been studied in the context of transcriptional regulation, little is known about how these interactions might affect DNA repair. Compared to the control cells, the shRNA knockdown cells were hypersensitive to ABT-888. The SF_70_ (dosage at which 70% of cells survived) was 100 μM for the control cells but only 3.5 μM for the RNAi knockdown cells (Fig. 2A). The SF_50_ was not reached in the control cells even at the highest dose (100 μM), which indicates ABT-888 was not very cytotoxic, while the SF_50_ was 25 μM for shRNA knockdown cells.

Inhibition of topoisomerase I can also result in SSBs being converted to lethal DSBs during DNA replication [38], so we hypothesized that the shRNA knockdown cells would also exhibit increased sensitivity to camptothecin. Similar to treatment with ABT-888, shRNA knockdown cells were hypersensitive to camptothecin. For camptothecin, the SF_50_ was 11.5 nM and 6 nM for the control cells and shRNA knockdown cells, respectively (Fig. 2B).

We performed NADPH assays, which provide a quantitative measurement of SSBs [39], and the shRNA knockdown cells did not have an increased number of SSBs either under basal conditions or in response to the alkylating agent methyl methane sulfonate (MMS) (Supplementary Fig. 1). This result suggests that the increased susceptibility of shRNA knockdown cells to PARP and camptothecin is not due to a higher number of SSBs to begin with but is instead due to an increased conversion of SSBs to DSBs.

Considering that cells deficient for SWI/SNF function appeared to be hypersensitive to genotoxic agents that increase the probability of generating DSBs at replication forks, we examined whether SWI/SNF complexes were required exclusively for survival from replication-dependent DSBs or DSBs in general. Interestingly, cells depleted of BRG1 and BRM were not hypersensitive to ionizing radiation (IR) (Fig. 2C) but were hypersensitive to etoposide (Fig. 2D), which inhibits topoisomerase II, an enzyme that induces DSBs to decatenate sister chromatids in S phase and G2 of the cell cycle. For etoposide, the SF_50_ was 490 nM and 125 nM for the control cells and shRNA knockdown cells, respectively (Fig. 2D).

We also subjected D98 cells to ultraviolet light-C (UV-C), which stalls DNA replication forks by inducing the formation of DNA lesions such as cyclobutane pyrimidine dimers and 6,4 photoproducts, and aphidicolin, which stalls DNA replication forks by inhibiting replicative DNA polymerases. Cells deficient for SWI/SNF complex activity were not hypersensitive to these replication fork-stalling agents (Fig. 2E and 2F). Lastly, we analyzed annexin V straining as an early marker of apoptosis and PI staining as a measure of DNA content and cell-cycle progression (Supplementary Fig. 2–3). UV, IR, and etoposide induced apoptosis by approximately 2-fold and altered the cell cycle within 1 hr of exposure, which is consistent with the genotoxicity of these agents.

### SWI/SNF complexes promote full activation of the DDR

**Figure 3:**
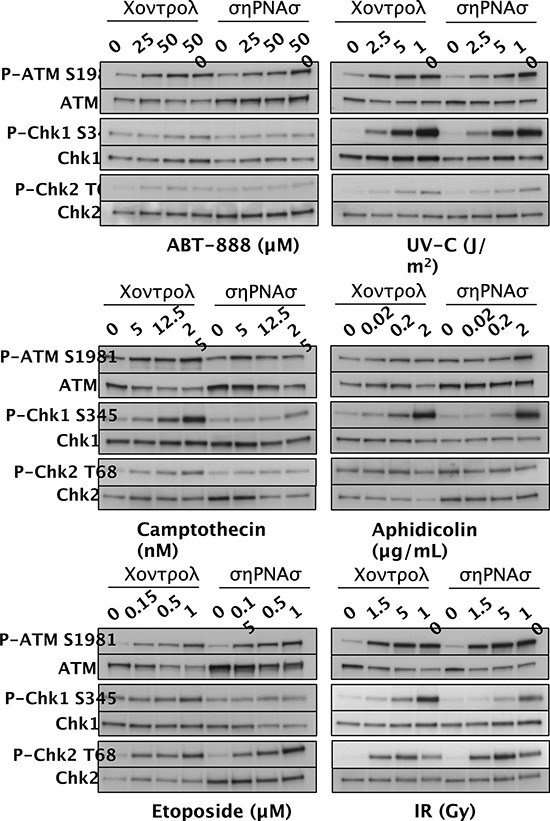
Activation of DDR proteins in D98 control cells versus shRNA knockdown cells (shRNAs) after exposure to genotoxic agents Western blots are shown for P-ATM S1981 and total ATM, P-Chk1 S345 and total Chk1, and P-Chk2 T68 and total Chk2. Genotoxic agents are shown below the western blot panels, and the doses are shown above each lane. The results are representative of 3 independent experiments.

**Figure 4:**
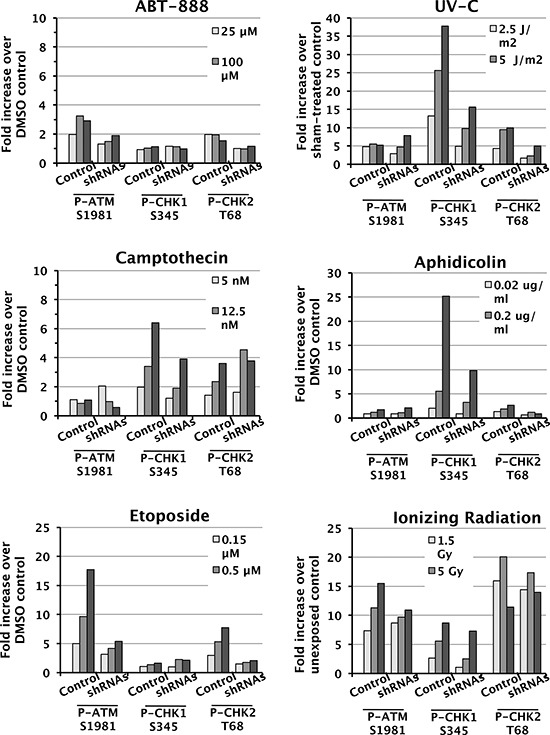
Attenuated induction of DDR protein phosphorylation in shRNA knockdown cells Quantification of P-Chk1 S345, P-ATM S1981, and P-Chk2 T68 levels normalized to total protein levels of each protein. The values correspond to the fold increase for each dose compared to vehicle-treated controls. Each panel corresponds to treatment with a different genotoxic agent (listed above) and shows controls (Cntrl) to the left and shRNA knockdown cells (shRNAs) to the right.

ATM and ATR are kinases that initiate complex signaling cascades in response to DNA damage [40], and Chk1 kinase, Chk2 kinase, and the histone variant H2AX are key phosphorylation targets of ATM and ATR. To determine whether SWI/SNF is required for the DDR, we assessed activation of the ATM-Chk2 and ATR-Chk1 signaling pathways. We exposed control D98 cells and shRNA knockdown cells to different doses of genotoxic agents and performed western blot analyses to detect P-ATM S1981, P-Chk1 S345, and P-Chk2 T68 (Fig. 3). The activated phospho-protein levels were normalized to total ATM, Chk1, or Chk2 and presented as their level of induction over control treatment alone (Fig. 4). As expected, each genotoxic agent stimulated the DDR in a dose-dependent manner, and P-ATM, P-Chk1, and P-Chk2 were induced to different extents depending on the genotoxic agent (Figs. 3 and 4). For example, aphidicolin induced P-Chk1 up to 25 fold but did not induce either P-ATM or P-Chk2, which is consistent with aphidicolin causing replication forks to stall and triggering the ATR arm of the DDR. On the other hand, IR induced P-ATM and P-Chk2 by up to 16 and 20 fold, whereas it induced P-Chk1 to a lesser extent (up to 8 fold). This is compatible with IR causing DSBs independent of DNA replication and primarily triggering the P-ATM arm of the DDR. These observations suggest that DDR activation is normal in D98 control cells. Overall, deficiency for SWI/SNF complexes reduced genotoxin-dependent activation of key DNA damage-response kinases by approximately 50%. However, SWI/SNF complexes did not appear to contribute to DDR activation in response to IR.

### SWI/SNF is required for maximal γH2AX levels in a genotoxin-specific manner

**Figure 5:**
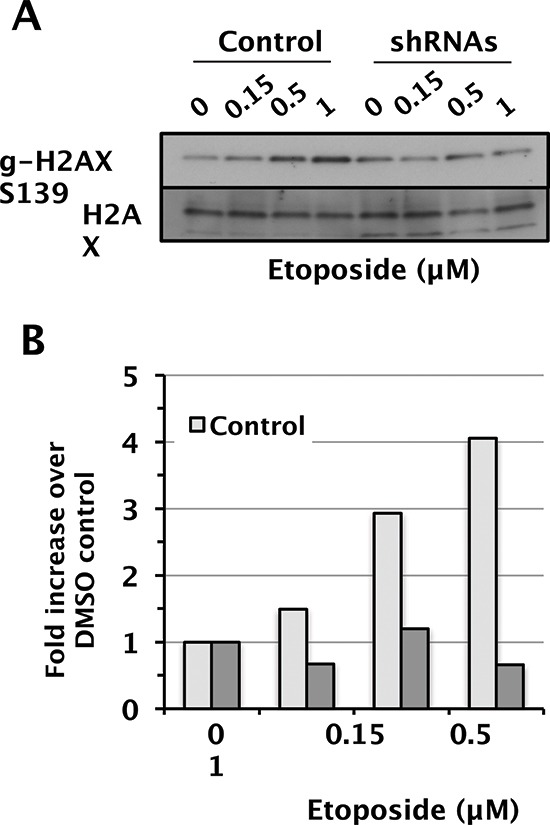
Attenuated induction γ-H2AX in D98 control cells versus shRNA knockdown cells after exposure to etoposide **(A)** Western blots showing γH2AX and total H2AX as a loading control. Etoposide doses are shown above each lane. **(B)** Relative levels of γH2AX normalized to total H2AX.

When ATM, ATR, and DNA-PK are activated, they can phosphorylate the histone variant H2AX at Ser139 (γH2AX) at damaged sites, which is an important, early event in the DDR. The BRG1 catalytic subunit has been implicated in γH2AX regulation [41, 42] so we compared γH2AX levels to total H2AX levels in the D98 control cells and shRNA knockdown cells. Camptothecin, etoposide, UV-C, and IR induced γH2AX in a dose-dependent manner that did not differ between control cells and shRNA knockdown cells (Supplementary Fig. 4). Surprisingly, however, induction of γH2AX was significantly attenuated in shRNA knockdown cells only in response to etoposide (Fig. 5A and 5B). The reason for this specificity is not known, but it does correlate with etoposide having the largest induction of P-ATM in control cells and the most attenuated P-ATM induction in shRNA knockdown cells. It is also consistent with recently reported links between SWI/SNF and topoisomerase II α (TOP2A) and topoisomerase II-binding protein 1 (TOPBP1) [17, 43].

### SWI/SNF is required for proper genome segregation

**Figure 6:**
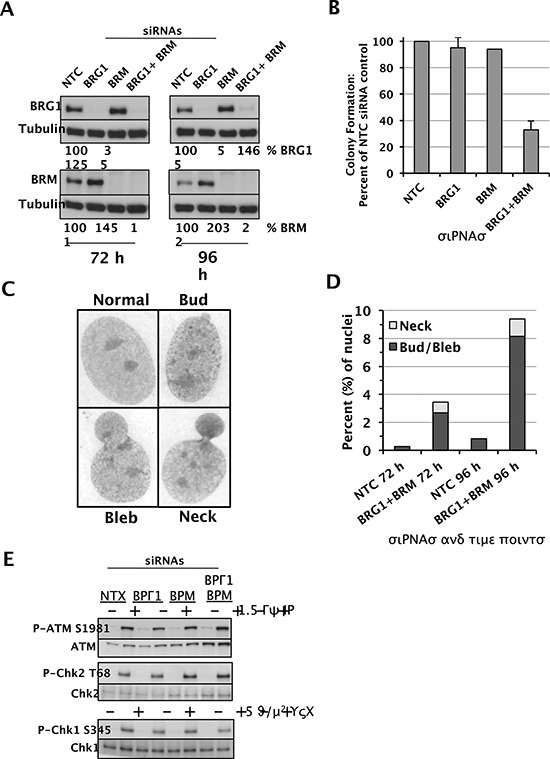
Depletion of the SWI/SNF catalytic subunits in NHF1-hTERT cells and functional outcomes **(A)** Western blot analysis of BRG1, BRM, and tubulin loading control in NHF1-hTERT cells transfected with the following siRNAs as indicated above each lane: NTC (non-targeted control), BRG1, BRM, or BRG1 and BRM simultaneously. Protein lysates were prepared 72 hours (left) or 96 hours (right) after electroporation of the siRNAs. Shown below each lane is the quantification of BRG1 and BRM protein levels normalized to tubulin. Protein levels in siBRG1 and/or siBRM cells are shown as a percentage of controls (which are set at 100). **(B)** Colony formation assays of NHF1-hTERT depleted of BRG1 or BRM, or both SWI/SNF catalytic subunits. Histograms show the mean ± standard deviation based on 3 independent experiments. **(C)** Representative images of normal nuclei and nuclei with buds, blebs, and necks. 100X magnification. **(D)** Quantification of nuclei with buds, blebs, and necks in NTC siRNA cells and double-knockdown cells (BRG1 + BRM siRNAs) at 72 hours and 96 hours after electroporation of siRNAs. **(E)** Activation of DDR proteins in NHF1-hTERT cells with RNAi-mediated knockdown of nontargeted control (NTC), BRG1, BRM, or BRG1 and BRM simultaneously. Western blots are shown for P-ATM S1981 and total ATM, P-Chk2 T68 and total Chk2, and P-Chk1 S345 and total Chk1. IR and UVC treatments are shown above each lane. The results are representative of 2–3 independent experiments.

One might expect an impaired DDR to result in genomic instability. Although the D98 knockdown cells grew slowly compared to controls, we did not observe chromosomal aberrations, micronuclei, or other nuclear anomalies at levels greater than the parental line (data not shown), presumably because this is a HeLa cell subline with an unusual karyotype that has mutations that preclude further genomic instability. Therefore, we depleted BRG1 and/or BRM in normal human fibroblasts (NHF1-hTERT), which have been immortalized with hTERT but are non-transformed and have normal karyotypes and an intact DDR and cell-cycle checkpoints [44, 45]. Western blot analyses confirmed a robust knockdown in each case and also revealed elevated BRM protein levels in siBRG1 cells that were approximately 150–200% relative to controls (Fig. 6A). The converse situation has long been known to occur, where BRG1 protein levels are upregulated in BRM-deficient cells [46], but this finding confirms a recent report that BRM can be upregulated in BRG1-deficient cells [47]. This finding suggests that BRG1 and BRM functionally compensate in NHF1-hTERT as they do in mouse embryos [48] and adult vascular endothelial cells [49]. Indeed, NHF1-hTERT depleted of BRG1 or BRM grew normally in colony forming assays, whereas cells depleted of both BRG1 and BRM formed colonies at only 30% of controls (Fig. 6B). Similar to the D98 cells, the double-knockdown NHF1-hTERT did not show signs of spontaneous chromosomal instability based on Giemsa-stained metaphase spreads (data not shown). However, they did show signs of aberrant nuclear morphology with the formation of buds, blebs, and necks (Fig. 6C). DAPI staining confirmed the presence of nucleoplasmic bridges in the neck structures (Supplementary Fig. 5). Quantification of these results demonstrated the presence of these aberrant nuclei in approximately 9% of double-knockdown cells, which was 10-fold higher than the control cells (Fig. 6D). However, despite this severe spontaneous phenotype, we did not observe an impaired DDR in double-knockdown NHF1-hTERT based on P-ATM and P-Chk2 expression in response to IR or P-Chk1 expression in response to UV-C (Fig. 6E).

## DISCUSSION

The results presented here demonstrate that SWI/SNF chromatin-remodeling complexes are required for the full activation of the DDR. In our experiments, the phosphorylation status of checkpoint proteins in the DDR was assessed 30–60 minutes after control or knockdown cells were exposed to genotoxic or fork-stalling agents. Such rapid kinetics strongly suggests that SWI/SNF plays a direct role at damaged sites rather than an indirect role meditated *via* transcriptional regulation of DNA repair factors. A direct role for SWI/SNF in the DDR is also supported by a proteomic screen that identified BRG1, BRM, and other SWI/SNF subunits as targets of ATM and ATR phosphorylation in response to IR or UV treatment [50]. Recently, a direct link between ATM and BRG1 phosphorylation was confirmed [51]. At a functional level, RNAi has been performed to demonstrate that the BAF60A subunit (also known as SMARCD1) is important for γH2AX induction and cell-cycle arrest in response to IR [50]. It also has been reported that BRM plays a role in the intra-S phase checkpoint as *Brm^−/−^*fibroblasts continue to undergo DNA replication and cell proliferation following IR [46]. A role for SWI/SNF in the DDR would explain why it has been implicated in multiple types of DNA repair including nucleotide excision repair, non-homologous end-joining, and homologous recombination [41, 42, 52–61]. It should be noted that some of these studies have reported functional differences, which is likely due to the comparison of cell lines derived from different tissue/tumor types that each have a unique genetic background. For example, BRG1 and BRM depletion had a much stronger effect on the survival of lung cancer cells [62] than what we observed for cervical cancer cells in this study.

In our experiments, SWI/SNF was important for full activation of ATM- and ATR-dependent responses of D98 cells to exogenous DNA damaging agents. This effect was also cell-type specific because SWI/SNF did not have the same effect in NHF1-hTERT cells. The DDR initiates signaling cascades that protect against replicative stress and genomic instability. Although we did not observe chromosomal aberrations in D98 cells or NHF1-hTERT depleted of BRG1 and BRM, we observed nucleoplasmic bridges in our knockdown NHF1-hTERT cells. These findings support previous observations where perturbation of the BRG1 catalytic subunit in ES cells, fibroblasts, and mammary epithelial cells resulted in aberrant nuclear morphology, anaphase bridge formation (in which sister chromatids are linked by catenated strands of DNA), micronuclei, and aneuploidy [43, 63–65]. A similar phenotype has been reported for SNF5-deficient mouse embryo fibroblasts (MEFs) [66]. DNA bridges can be severed during cytokinesis [67], which often results in partial or complete chromosomal gains or losses. Segregation defects also occur *in vivo* based on the extensive copy number gains and losses that were previously observed by array CGH in mammary tumors from our *Brg1* mouse model of breast cancer [11]. The cause of aberrant nuclear morphology in cells deficient for SWI/SNF catalytic subunits remains to be fully characterized, but it could be due in part to attenuation of the DDR.

Our results combined with these previous studies support the idea that SWI/SNF tumor suppression involves the maintenance of genomic stability. A recent study proposed a model for the molecular mechanism where SWI/SNF physically interacts with TOP2A and that BRG1 ATPase activity is required for TOP2A to bind chromatin [43]. This is compatible with our observation that SWI/SNF is required for the activation of ATM in response to etoposide but not other genotoxic agents. This model is also consistent with our previous findings that BRG1 co-immunoprecipitates (co-IPs) with TOPBP1 [17]. A link between SWI/SNF and TOP2A function might also be expected to apply to normal DNA replication during development. This is certainly the case for BRG1 and SWI/SNF, which are required for efficient replication fork progression and embryonic survival [10, 17], and is undoubtedly true for TOP2A function as well. Although *Top2a* knockout mice have not been described, *Topbp1* knockouts die at the same early stage of embryogenesis as *Brg1* null homozygotes [68], and TOPBP1 is involved in normal DNA replication as well as cancer prevention [69].

An unexpected but important aspect of our study is that SWI/SNF-deficient cells are specifically hypersensitive to PARP and topoisomerase inhibitors that induce the formation of highly supercoiled DNA between a frozen topoisomerase enzyme and a replication complex. This common feature, which is not shared by any of the other genotoxic agents that were utilized, suggests that SWI/SNF chromatin remodeling is required to bypass these structures. This might represent a major mechanism of SWI/SNF in the DDR. Lastly, the results of our cytotoxicity experiments suggest that knowing the status of SWI/SNF functionality in tumors could allow for better selection of anticancer chemotherapeutic treatment. These findings suggest that SWI/SNF mutant tumors will respond better to PARP and topoisomerase inhibitors than cisplatin or IR in the clinical setting.

## METHODS

### Cell culture

The D98 HeLa knockdown cells, which have marked reductions of BRG1 and BRM, have been described and validated previously [17]. Briefly, the cells were grown under puromycin (2 μg/ml) and G418 (1 μg/ml) selection in RPMI supplemented with 10% FBS to maintain expression of stably integrated *BRG1* and *BRM* shRNA constructs, respectively. NHF1-hTERT cells were cultured in Dulbecco's modified Eagle's medium supplemented with 2 mM L-glutamine and 10% fetal bovine serum. Cell culture reagents were obtained from Sigma-Aldrich, and cells were grown at 37°C in a humidified atmosphere of 5% CO_2_.

### Exposure to genotoxic agents

For exposure to UV-C, cell culture medium was reserved, cells were washed with 37°C PBS, PBS was aspirated, and cells were exposed to UV-C or placed into the exposure chamber without exposure (control). Reserved cell culture medium was returned to the dishes and cells were incubated for 45 min before harvest. Cells were exposed to ionizing radiation using an RS2000 Biological Irradiator (Rad-Source). Cells were harvested 30 min after exposure. Cells were exposed to chemicals for one hour and concurrent controls exposed to the appropriate solvent were used for each experiment. The concentration of DMSO in cell culture medium did not exceed 0.1%.

### D98 cell survival assays

D98 cell survival was assessed in triplicate in the absence of selection by plating ~2.5 × 10^3^ cells in 250 μL per well in 24-well plates and exposing to ABT-888, camptothecin, etopocide, cisplatin, MMS, UV-C, aphidicolin, or IR. After 3 days, cells were cultivated and viability was determined by the XTT assay [70, 71].

### Western blot analyses

At the time of harvest, cell culture medium was aspirated, cells were washed with PBS, and were detached from dishes by trypsin. Trypsinization was stopped with medium containing 10% FBS, and cells were spun down and washed once with ice-cold PBS. Cells were counted using a Coulter counter (Beckman Coulter) and re-suspended in a volume of 2X SDS lysis buffer (62.5 mM Tris-Cl pH 6.8, 2% SDS, 25% glycerol, and 0.01% bromophenol blue with 10% β-mercaptoethanol added at the time of use) for a concentration of 1 million cells/100 μl buffer. Protein lysates were boiled for 10 min and stored at −80°C. Equal volumes of protein lysates were run per well in pre-cast protein electrophoresis gradient gels (BioRad) and proteins were transferred onto nitrocellulose membranes (BioRad). Membranes were blocked with 5% BSA (for detection of phospho-proteins) or 5% milk before application of primary antibodies. The antibodies used in this study were rabbit anti-BRM (Abcam, ab15597), mouse anti-BRG1 (Santa Cruz Biotechnology, sc-17796/G7), mouse anti-α-tubulin (Sigma, T6199, rabbit anti-P-ATM S1981 (Epitomics, EP1890Y), rabbit anti-P-Chk2 (Cell Signaling, 2661), rabbit anti-P-Chk1 S345 (Cell Signaling, 2348), mouse anti-γ-H2AX S139 (Millipore, 05–636), rabbit anti-ATM (Bethyl, A300-299A-2), mouse anti-Chk2 (BD Biosciences, 611570), mouse anti-Chk1 (Santa Cruz, sc-8408), and rabbit anti-H2AX (Millipore, 07–627). When applicable, membranes were probed first with phospho-antibodies, stripped, and re-probed for total protein. Image J software was used to quantify signals (Rasband, WS, Image J US National Institute of Health, Bethesda, MD, http://rsb.info.nih.gov/ij/). Levels of BRG1 or BRM were normalized to α-tubulin, and phospho-proteins were normalized to their respective total protein levels.

### Protein depletion by siRNA

NHF1-hTERT were electroporated with siRNAs using the normal human dermal fibroblast nucleofection kit VPD-1001 (Lonzo) and electroporation program U-23. The total amount of siRNA introduced into cells for single versus double depletions was held constant at 200 pmol siRNA per 1 million cells (100 pmol of targeting siRNA was combined with 100 pmol of NTC siRNA for single depletions). siGENOME Smart Pool siRNAs were purchased from Dharmacon to deplete BRG1 (M-010431) or BRM (M-017253). Non-targeted control (NTC) siRNA (D001206) was also obtained from Dharmacon.

### Clonogenic survival assays

The clonogenic survival assays were performed as previously described [72]. Briefly, NHF1-hTERT cells electroporated with siRNAs were seeded at a density that would result in ~150 colonies per 10 cm dish for the NTC siRNA control. Each independent experiment was seeded in triplicate, and the experiment was repeated two to three times per siRNA. Cells were stained in a solution of 0.05% Crystal Violet in 40% methanol on day 14 after seeding. Colonies of 50 cells or more were counted.

### Metaphase preparations

Giemsa-stained metaphases were prepared as previously described [72]. Briefly, 50 metaphases with bifilar sister chromatids were evaluated per treatment. For measures of nuclear abnormalities, approximately 1,000 nuclei were examined per treatment. The experimenter was blind to treatment during the collection of data and analysis of chromosomal aberrations and nuclear abnormalities.

## SUPPLEMENTARY FIGURES


