# Supplementary material for: SWI/SNF complexes are required for full activation of the DNA-damage response
Source: Oncotarget. 2015 Jan 6;6(2):732–45. doi: 10.18632/oncotarget.2715 (PMC4359251; doi:10.18632/oncotarget.2715)
Supplement: Supplementary file 1 [file oncotarget-06-732-s001.pdf]

SUPPLEMENTARY FIGURES

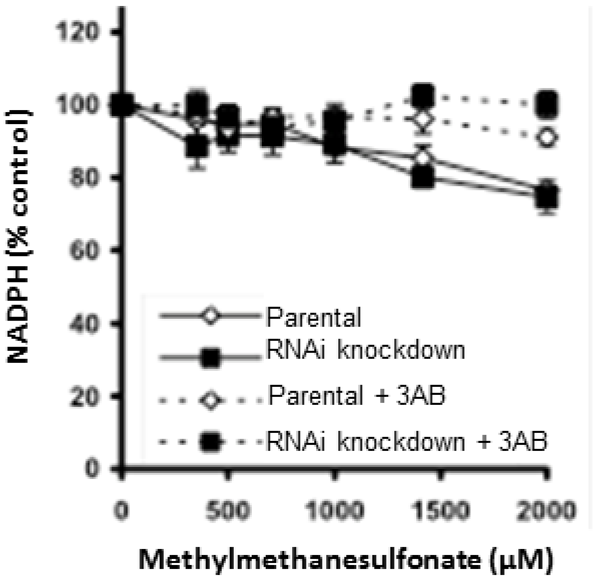

Supplementary Figure 1: NADPH levels of D98 control cells (open shapes) and RNAi knockdown cells (filled shapes) after being exposed to MMS. + 3AB, 3-aminobenzamide inhibits the ligation of MMS-induced SSBs and served as a control. Results are shown as the mean ± SE for three independent experiments.

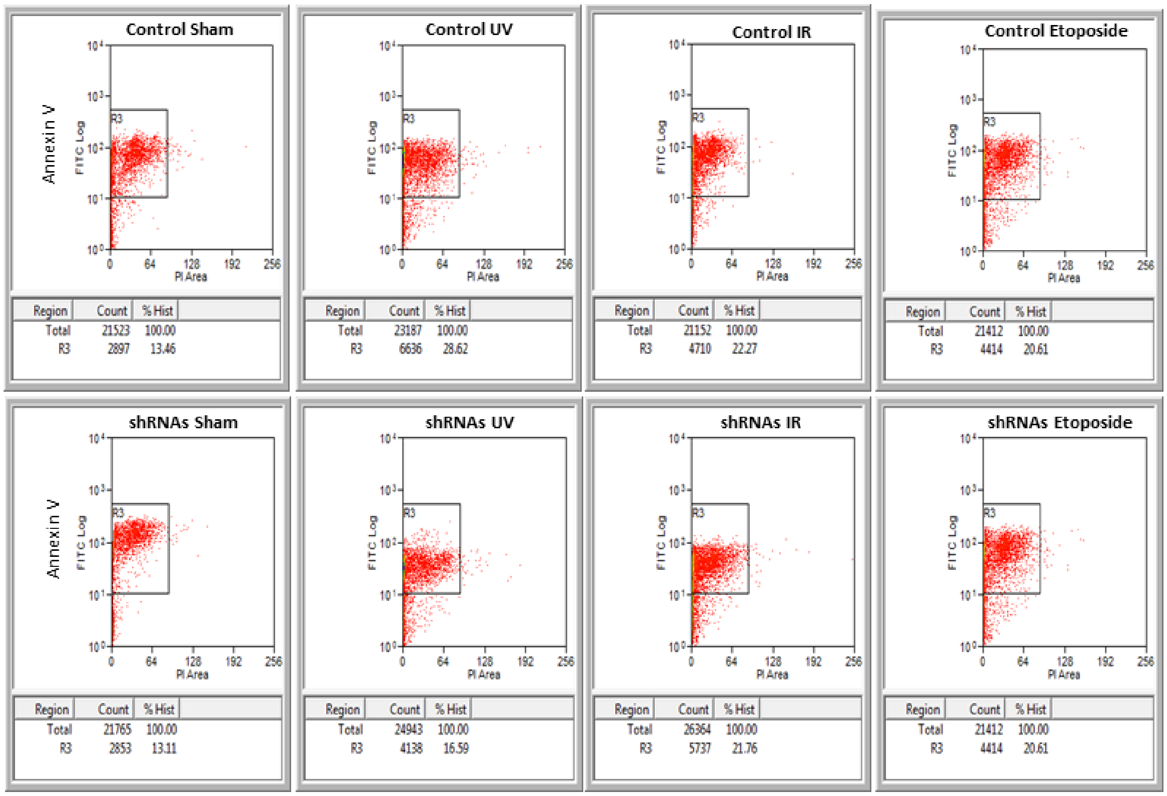

Supplementary Figure 2: Characterization of apoptosis in D98 control cells (top panels) and cells depleted of BRG1 and BRM (shRNAs, bottom panels) under sham conditions (far left) and in response to UV (10 J/m^2), IR (10 Gy), and etoposide (0.5 µM). Representative flow cytometric scatter plots are shown with annexin V on the y-axis and propidium iodide (PI) on the x-axis. Annexin V-positive cells are quantified in the R3 box in each panel. There are relatively few PI-positive cells because cells were analyzed 1 hr after each exposure.

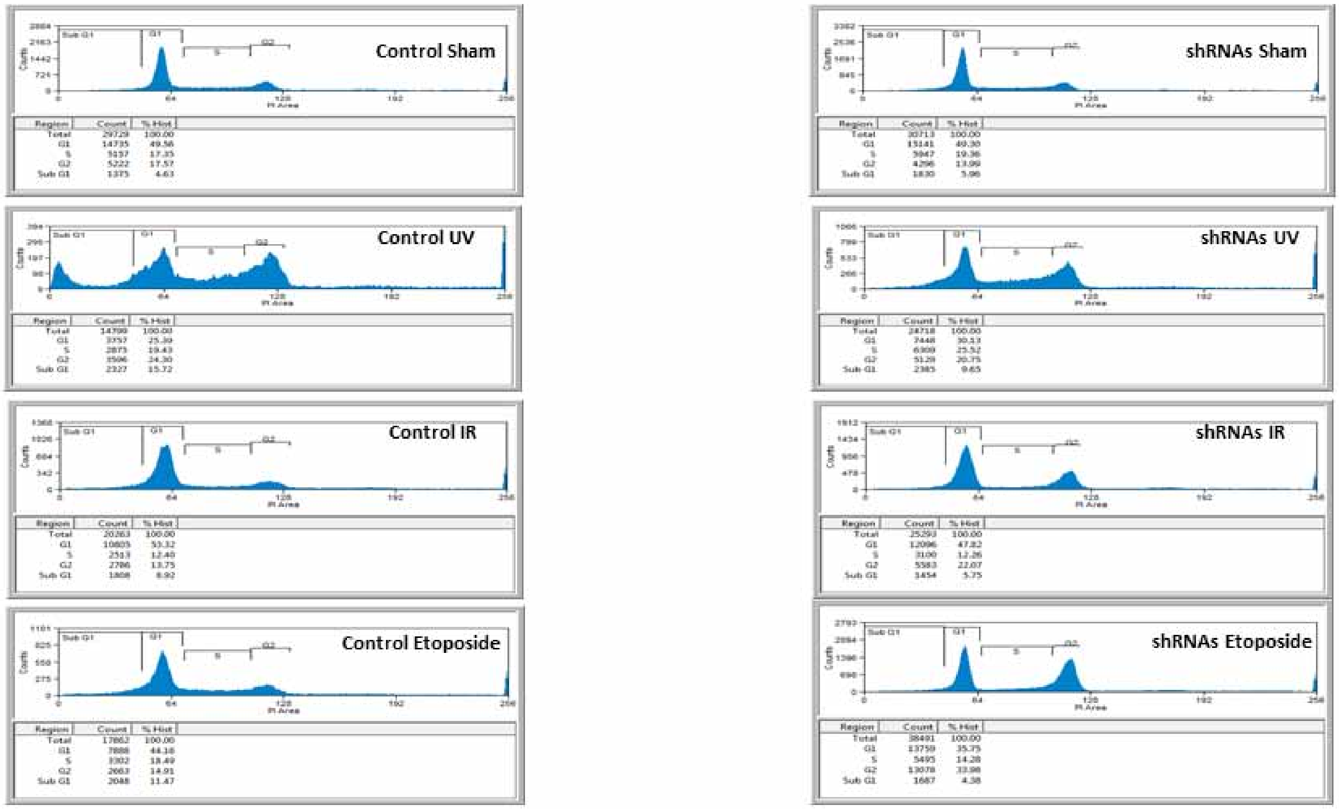

Supplementary Figure 3: Characterization of cell cycle in D98 control cells (left panels) and cells depleted of BRG1 and BRM (shRNAs, right panels) under sham conditions (top panels) and in response to UV (10 J/m^2), IR (10 Gy), and etoposide (0.5 µM). Representative flow cytometry plots of propidium iodide (PI) as a measure of DNA content are shown. The following populations are labeled in each panel: Sub G1, G1, S, G2 (which includes M).

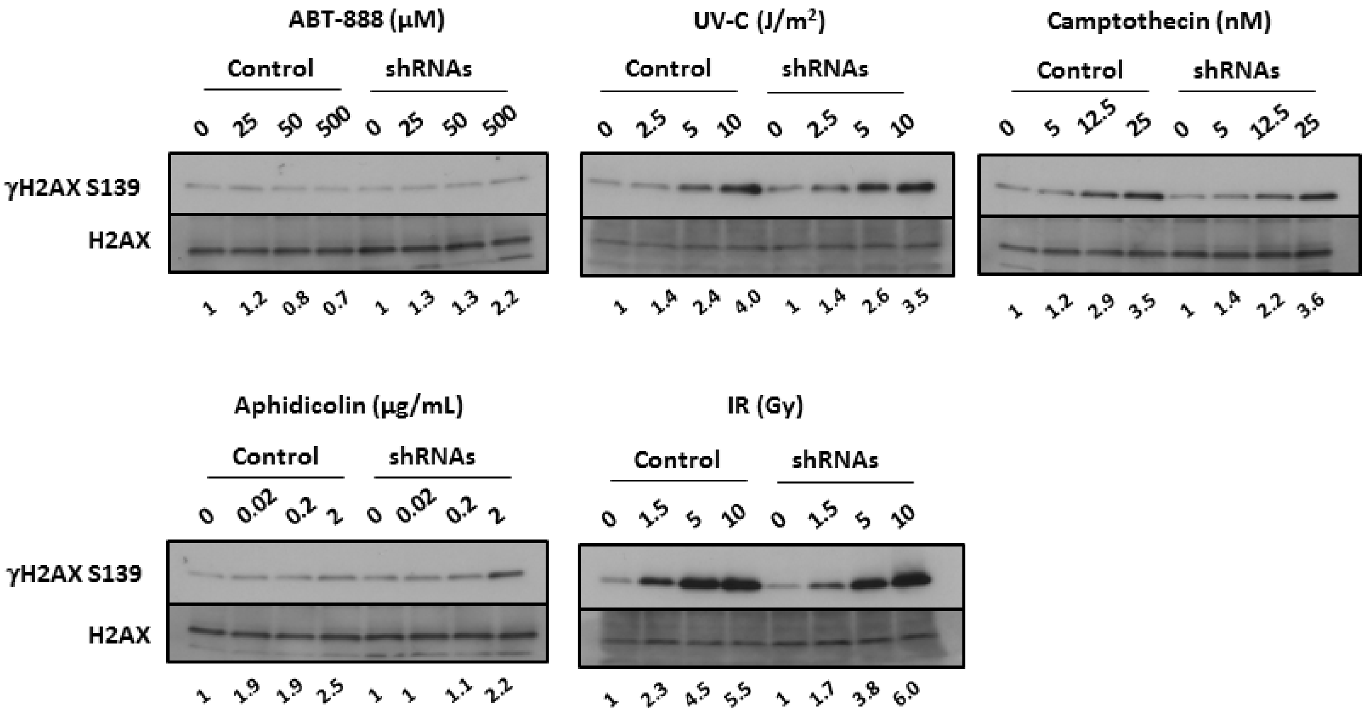

Supplementary Figure 4: Induction of γ-H2AX by various genotoxic agents (other than etoposide, see Figure 5) is not affected by knockdown of SWI/SNF catalytic subunits in D98 cells. Western blots showing γ-H2AX and total H2AX as a loading control. Genotoxins and their doses are shown above each lane. γ-H2AX was first normalized to total H2AX and the numbers below the western blots report the fold increase in normalized γ-H2AX compared to the treatment control.

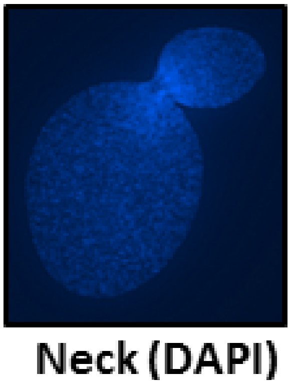

Supplementary Figure 5: Representative DAPI image neck in NHF1 cells simultaneously depleted of BRG1 and BRM to show presence of DNA in nucleoplasmic bridge.
